# Molecular Mechanisms of Hypothalamic Insulin Resistance

**DOI:** 10.3390/ijms20061317

**Published:** 2019-03-15

**Authors:** Hiraku Ono

**Affiliations:** Department of Endocrinology, Hematology and Gerontology, Chiba University Graduate School of Medicine, Chiba 260-8670, Japan; hono@chiba-u.jp; Tel.: +81-43-226-2091

**Keywords:** hypothalamus, insulin resistance, inflammation, obesity, food intake, glucose metabolism

## Abstract

Insulin exists in the central nervous system, where it executes two important functions in the hypothalamus: the suppression of food intake and the improvement of glucose metabolism. Recent studies have shown that both are exerted robustly in rodents and humans. If intact, these functions exert beneficial effects on obesity and diabetes, respectively. Disruption of both occurs due to a condition known as hypothalamic insulin resistance, which is caused by obesity and the overconsumption of saturated fat. An enormous volume of literature addresses the molecular mechanisms of hypothalamic insulin resistance. IKKβ and JNK are major players in the inflammation pathway, which is activated by saturated fatty acids that induce hypothalamic insulin resistance. Two major tyrosine phosphatases, PTP-1B and TCPTP, are upregulated in chronic overeating. They dephosphorylate the insulin receptor and insulin receptor substrate proteins, resulting in hypothalamic insulin resistance. Prolonged hyperinsulinemia with excessive nutrition activates the mTOR/S6 kinase pathway, thereby enhancing IRS-1 serine phosphorylation to induce hypothalamic insulin resistance. Other mechanisms associated with this condition include hypothalamic gliosis and disturbed insulin transport into the central nervous system. Unveiling the precise molecular mechanisms involved in hypothalamic insulin resistance is important for developing new ways of treating obesity and type 2 diabetes.

## 1. Introduction

Obesity is a common problem worldwide, as it contributes to type 2 diabetes and other life style-related diseases in susceptible people with genetic predispositions. In addition to the easy access to calorie-dense foods and the predominance of lifestyles with little or no physical exercise in modern society, a common cause of obesity is the lack of effective drugs against obesity that are free of unacceptable side effects [1]. Exploring medications that are effective in treating obesity by suppressing food intake and/or enhancing energy expenditure is among the most important research goals in modern medicine.

Insulin, the pancreatic hormone secreted to maintain normal blood glucose levels, has been recognized to suppress food intake and weight gain when injected into cerebral ventricles [2]. More recently, insulin has been found to improve peripheral glucose metabolism in the brain [3], independent of its effects on food intake and body weight. Therefore, targeting insulin in the brain could be a valid approach for treating obesity and type 2 diabetes, provided that its functions in the brain remain intact. These beneficial effects are severely disturbed by excessive nutrition, the consumption of fatty foods, and obesity itself, a condition referred to as brain insulin resistance. Obesity induces brain insulin resistance, which blunts the suppressive action of insulin on food intake, thus inducing more severe obesity. In other words, a vicious cycle develops and persists between obesity and brain insulin resistance. Therefore, clarifying the mechanism by which brain insulin resistance occurs, and devising strategies for breaking this vicious cycle, are important for developing new medications for the effective treatment of obesity and type 2 diabetes.

## 2. Two Major Insulin Functions in the Hypothalamus: Suppression of Food Intake and Endogenous Glucose Production

When insulin is injected into the cerebral ventricles of rodents, food intake [2] and endogenous glucose production are both suppressed [4]. When insulin is sprayed into the nostrils of humans, food intake [5] and endogenous glucose production [6] are both suppressed. Brain-specific insulin receptor (IR)-knockout (NIRKO) mice are an animal model of both obesity and insulin resistance [7]. Deletion of IR in the hypothalamus using an antisense oligonucleotide induced hyperphagia and insulin resistance [8]. These data consistently demonstrate that insulin in the central nervous system (CNS) stimulates insulin signaling in some hypothalamic cell types, thereby suppressing food intake and regulating glucose metabolism.

However, the question remains as to which cell types in the hypothalamus are involved in these effects. In NIRKO, where Cre recombinase is driven by nestin, IR is deleted in neurons and glial cells, suggesting that insulin may act on both cell types by transducing their effects [9]. Neurons are an intensively studied cell type, and studies have shown that insulin signaling initiated by insulin receptor (IR) activation ultimately results in electrophysiological and/or transcriptional changes in neurotransmitters that are within or released by neurons. More recent studies have also revealed the involvement of non-neuronal cells. Most notably, insulin was found to work in astrocytes, transporting glucose from peripheral blood into the CNS [10,11]. Astrocyte-specific deletion of IR disturbed glucose sensing in the hypothalamus, resulting in impaired glucose tolerance and systemic insulin resistance [11]. Moreover, insulin receptors on vascular endothelial cells are reportedly involved in insulin transport from the periphery to the brain [12,13,14]. Tanycytes, a special cell type lining the third ventricle, have recently attracted attention as being responsible for the transport of hormones and nutritional signals crossing the blood–brain barrier (BBB) [15]. While tanycytes have been shown to transport leptin [16,17] via its receptor, the role of these cells in insulin transport requires further study.

The most important point regarding the effects of insulin on food intake and glucose metabolism is that these functions are not always independent of each other. If blocking hypothalamic insulin signaling induces significant changes in food intake—which would chronically result in obesity or leanness—then glucose metabolism would be impaired or improved due to the resulting obesity and leanness, respectively. This could lead to misunderstanding the primary effects on glucose metabolism. Therefore, the primary effect of intervening in hypothalamic insulin signaling on glucose metabolism can be demonstrated only by: (1) The lack of a significant effect on body weight; (2) an acute-phase intervention such as 1–3 days of a high fat diet (HFD), during which it is still too early for obesity to occur; or (3) food restriction in the orexigenic or to-be-obese group to match body weights between groups (pair-feeding).

**Figure 1:**
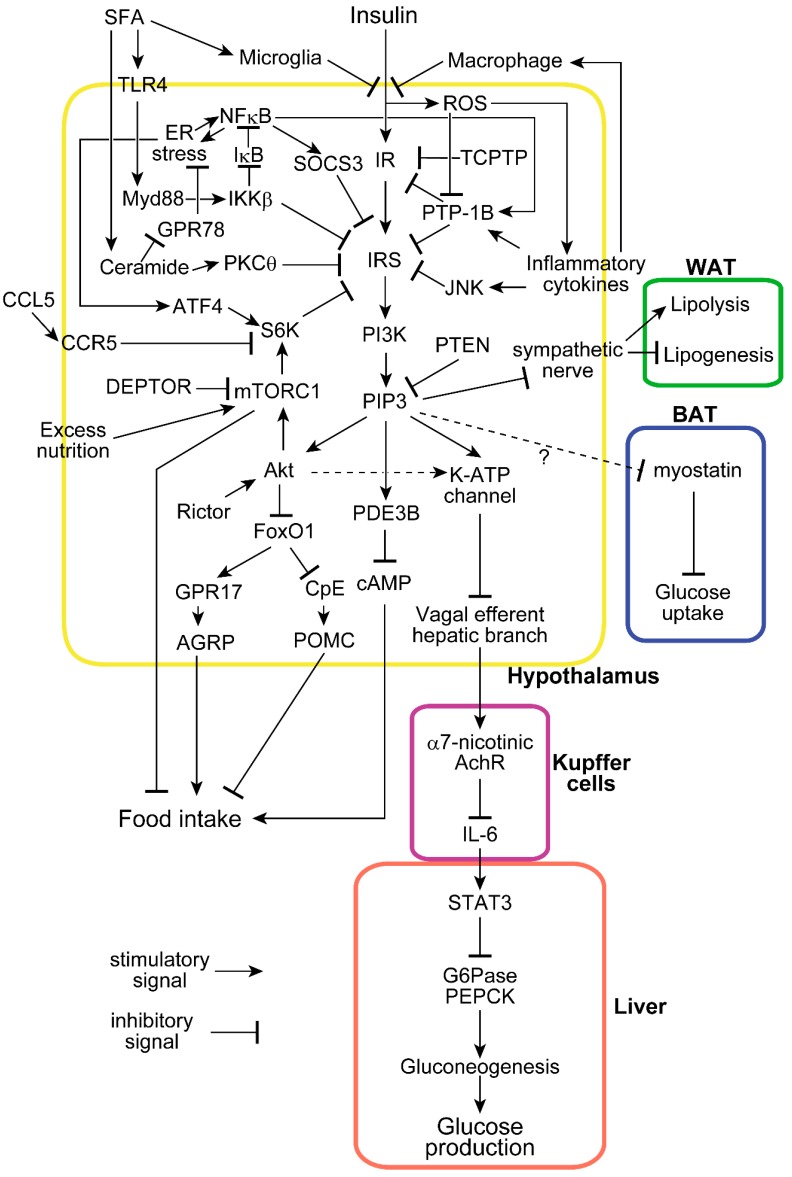
Molecular mechanisms of insulin functions and associated modifications in the hypothalamus. Insulin enters the hypothalamus and suppresses food intake and hepatic glucose production. Many molecules disturb hypothalamic insulin signaling at several sites in response to saturated fatty acids (SFA), inflammation, and excess nutrition. Hypothalamic insulin also affects metabolic processes in white adipose tissue (WAT) and brown adipose tissue (BAT). The dot line shows a pathway less confirmed, and the dot line with “?” is a hypothetical unconfirmed pathway.

The molecular mechanism by which insulin signaling in the hypothalamus suppresses food intake and mediates systemic glucose metabolism has been intensively studied [18]. Specific IR tyrosine residues are phosphorylated by IR itself when it binds insulin, thereby inducing tyrosine phosphorylation of insulin receptor substrate (IRS) proteins. This process results in the activation of PI 3-kinase, which in turn produces phosphatidylinositol (3,4,5) triphosphate (PIP3) [19]. When inhibitors of PI 3-kinase are injected into the ventricle, insulin neither suppresses food intake [20] nor enhances glucose metabolism [21], showing that PI 3-kinase activation is necessary for both effects of central insulin. We bidirectionally modulated PTEN, the negative regulator of PI 3-kinase signaling, in the rat hypothalamus and showed that hypothalamic PIP3 is responsible for the regulation of food intake and glucose metabolism [22]. These studies indicate that the effects on both food intake and glucose metabolism occur via a common pathway from IR to PI 3-kinase (Figure 1).

Elevated PIP3 induces Akt phosphorylation and activation in insulin-sensitive tissues. Neuronal-specific deletion of Rictor, the key component of mTORC2, which activates Akt by phosphorylating its serine 473 residue, induces obesity and impairs glucose tolerance [23]. Consistently, the proopiomelanocortin (POMC) neuron-specific deletion of Rictor enhances food intake [23]. Among Akt’s many substrates, transcription factor FoxO1 is phosphorylated by Akt and inactivated by nuclear exclusion upon insulin stimulation. Without insulin, FoxO1 transcriptionally increases orexigenic neuropeptide AGRP via GPR17 [24] and decreases anorexigenic neuropeptide POMC via carboxypeptidase E (CpE) [25]. Therefore, insulin presumably suppresses food intake via the IR–IRS–PI3k–PIP3–Akt–FoxO1–GPR17–AGRP/CpE–POMC pathway. Notably, deleting hypothalamic FoxO1 via the Nkx2.1 promoter only resulted in a mild food intake- and glucose metabolism-related phenotype, implicating extrahypothalamic FoxO1 in the observed effects on neuropeptides [26]. Furthermore, the role of hypothalamic Akt itself in food intake has yet to be fully explored, making it a topic for future investigations. On the other hand, the effects of insulin on glucose metabolism are mediated by the ATP-sensitive potassium (K^ATP^) channel [3], which is activated by PI 3-kinase and its downstream molecule, PIP3. Hypothalamic activation of the K^ATP^ channel transduces this signal to Kupffer cells in the liver via electrical inactivation of the hepatic branch of the efferent vagus nerve [4]. Acetylcholine signaling via the α7-nicotinic acetylcholine receptor (α7nAchR) in Kupffer cells is suppressed when hypothalamic insulin signaling is activated and the hepatic branch of the vagus is electrically suppressed, resulting in the stimulation of hepatic IL-6/STAT3 signaling, which ultimately transcriptionally suppresses key gluconeogenic enzymes and hepatic glucose production [27]. Therefore, insulin suppresses hepatic glucose production via the IR–IRS–PI3k–PIP3–K^ATP^ channel–vagal efferent-α7nAchR–IL6–STAT3 pathway. While the K^ATP^ channel is thought to be downstream of PIP3 but not Akt, recent findings have indicated that Rictor deletion in AGRP neurons induces mild glucose intolerance without changing body weight, indicating that at least a portion of hypothalamic insulin’s glucose-regulatory mechanism is located downstream of Akt [23].

Phosphodiesterase-3B (PDE3B), another mediator of insulin signaling in the hypothalamus, has also been recently identified. Intra-cerebro-ventricular (ICV) insulin activates PDE3B. Hypothalamic inactivation of PDE3B by a specific inhibitor or a genetic deletion blunts central insulin-induced anorexia or weight gain, respectively [28,29]. PDE3B is activated by PI 3-kinase and decreases the intracellular cAMP level [30], which is another potential pathway by which insulin suppresses food intake by acting on the hypothalamus. Interestingly, while PDE3B is downstream from PI 3-kinase, it is independent of Akt phosphorylation [31].

Hypothalamic insulin has recently been shown to be involved not only in glucose, but also in fat metabolism. Insulin acts on POMC neurons to suppress lipolysis, thereby enhancing lipogenesis in adipose tissue, which transduce the signal via the sympathetic nerves [32,33]. Moreover, the acute activation of AGRP neurons, by optogenetic or designer receptors exclusively activated by designer drugs (DREADD) technologies, has been shown to suppress glucose uptake in brown adipose tissue by upregulating myostatin [34]. While it is not clear whether this effect depends on hypothalamic insulin, taken together with the observation that insulin inactivates AGRP neurons, the mechanism by which hypothalamic insulin improves systemic glucose metabolism may involve the suppression of liver glucose production and glucose uptake into brown adipose tissue.

While the hypothalamus has been most intensively studied as an insulin-sensitive site involved in regulating food intake and glucose metabolism in the CNS, extrahypothalamic regions have also been implicated. Nasal insulin administered to human’s decreases blood flow in the hypothalamus and prefrontal cortex, a phenomenon that is blunted in overweight people [35]. Since the prefrontal cortex has a crucial role in decision making, including feeding behavior, this area may be an extrahypothalamic target by which insulin regulates food intake. Moreover, food palatability is reduced by CNS insulin via suppression of mesolimbic pathways in both human [36] and animal [37].

However, the central effects of insulin should not be overstated. To our knowledge, there have been no studies showing that CNS insulin exerts effects strong enough to induce hypoglycemia, indicating that its effect on glucose metabolism of central insulin is relatively moderate, and presumably easily compensated for by counter-regulatory hormones. Several animal studies using super-physiological doses of insulin have demonstrated its suppressive effects on food intake [38]. Inconsistent results regarding the effects of insulin on food intake have been obtained using animal models [39]. Central insulin suppression of hepatic glucose production has not been observed in dogs [40]. The effects of intranasal insulin administration in humans remain controversial due to its spillover into the bloodstream [41]. These observations indicate that further intensive studies are required to clarify the roles of species and timing that would provide robustly beneficial effects of central insulin [42,43].

Compared to other insulin-sensitive tissues such as the liver, muscle, and adipose tissue, it is interesting that the hypothalamus uses a common proximal signaling cascade from the IR to PI 3-kinase/Akt for glucose metabolism regulation. However, the distal signaling pathways are more specific to each tissue. Furthermore, the existence of the BBB makes insulin transport a unique potential blockage point for the CNS when considering insulin resistance in target tissues.

## 3. Hypothalamic Insulin Resistance Induced by Excessive Nutrition

While administering insulin as a nasal spray suppresses food intake and endogenous glucose production in normal-weight humans, these phenomena are lost in obese individuals [6,44]. Similarly, while nasal insulin suppresses hypothalamic blood flow in lean people, as demonstrated by functional magnetic resonance imaging, this suppression is blunted in obese individuals [35]. This observation of “brain insulin resistance” has been mirrored in rodent studies: ICV insulin does not suppress food intake in high-fat-diet (HFD)-fed rats [45], and even a single day of HFD abolishes the suppressive effects of hypothalamic insulin on hepatic glucose production [46]. These data show that both beneficial effects of central insulin are disturbed by obesity. An important area of research focuses on which part of the insulin transport and/or signaling pathway is blocked in the hypothalamus. Some studies have indicated that insulin delivery from the bloodstream to the CNS is disturbed by HFD feeding [12,13]. However, higher insulin concentrations—even in the cerebrospinal fluid (CSF) in obese individuals [47,48,49]—indicate the involvement of mechanisms other than disturbed insulin delivery to the CNS. Several studies have shown a decrease in tyrosine phosphorylation of IR and IRS [46,50], which can be partly explained by the increase in two tyrosine phosphatases, PTP-1B [51] and TCPTP [52,53], which are detailed below. However, only long-term, i.e., not short-term, HFD feeding has been shown to increase both phosphatases. Another mechanism underlying the decrease in the tyrosine phosphorylation of IRS-1 involves the serine phosphorylation of this protein, which inhibits the former. P70 S6 kinase [46] and JNK [54] are known to phosphorylate the serine residues of IRS-1, mediating the inhibition of insulin signal transduction. In our study, the suppression of hypothalamic PTEN in HFD-fed rats reversed insulin resistance without exerting effects on food intake [22], indicating that the HFD-induced blockage of hypothalamic insulin signals, such as IRS-1 serine phosphorylation, exist upstream from PI 3-kinase. Another blockage point exists downstream PI 3-kinase and impacts the regulation of food intake. Insulin activates the hypothalamic K^ATP^ channel in lean but not obese rats [55], showing that a site between PIP3 and the K^ATP^ channel may be blocked by HFD feeding. Such blockages impact the anorexic effect of hypothalamic insulin.

How rapidly does this hypothalamic insulin resistance occur? Hypothalamic insulin resistance caused by excessive nutrition occurs more rapidly than that in other insulin-sensitive tissues. Three days of HFD feeding sufficiently blunt the suppressive effects of insulin on food intake in rats [56]. We found that one day of HFD feeding was enough to blunt the suppressive effects of hypothalamic insulin on glucose production [46]. This one-day HFD also decreased tyrosine phosphorylation of IRS-1 and Akt phosphorylation in the hypothalamus, but not in the liver. Hepatic insulin resistance, which blunts endogenous glucose production, occurred within three days of initiating HFD feeding, while insulin resistance developed later in muscle and adipose tissues [57]. In contrast, hepatic insulin signaling, such as that involving PI 3-kinase activity, is somewhat upregulated by HFD feeding [58]. This controversy is explained by blunting the hypothalamic pathway, which potentially induces compensatory upregulation of hepatic insulin signaling [59].

## 4. Inflammation with ER Stress Induces Hypothalamic Insulin Resistance

Inflammation is an important pathway responsible for hypothalamic insulin resistance [60]. Inflammation induces biphasic effects on food intake. While high-level inflammation such as adenovirus infection in the hypothalamus [22] or ICV injection of high dose TNFα [61] suppresses food intake, low-level inflammation induced by ICV injection of low dose TNFα instead blocks the anorexic effects of ICV insulin as well as insulin signaling in the hypothalamus [61]. It is conceivable that after severe inflammation associated with a life-threatening infection, systemic recovery is permitted by higher nutritional intake, which would be evolutionarily programmed as an orexigenic reaction caused by low-grade inflammation. Long-chain saturated fatty acids (SFAs) cross the BBB, accumulate in the hypothalamus [45], and induce acute hypothalamic inflammation via microglial activation [62,63]. SFA binds to Toll-like receptor 4 (TLR4) [64], activates the IKKβ/NFκB pathway by activating the myeloid differentiation primary response gene 88 (MyD88) [64,65], and ultimately enhances the expressions of pro-inflammatory genes such as TNFα, IL-1β, and IL-6 in the hypothalamus [45,50].

Endoplasmic reticulum (ER) stress is a condition in which the ER cannot carry out normal protein folding and assembly. ER stress is also responsible for hypothalamic insulin resistance [66]. Glucose regulated protein 78 kDa/binding immunoglobulin protein (GPR78/Bip) has recently been found to reverse ceramide-induced hypothalamic ER stress [67]. SFA-induced ER stress [64] in the hypothalamus also contributes to activation of the IKKβ/NFκB pathway [68].

It is not caloric excess but rather SFA that initiates this inflammation signal, because even when the same number of calories in a fat-rich diet and normal chow are provided, activation of the IKKβ/NFκB pathway can still be observed [45]. In contrast, a recent finding that excess carbohydrate, not fat, induces hypothalamic inflammation via advanced glycation end products [69] challenges the canonical SFA-induced inflammation theory. Unlike SFA, unsaturated fatty acids such as oleic acid in the hypothalamus exert a relatively anorectic effect and enhance insulin sensitivity [70].

Another inflammation pathway starting from TLR4 activation is the MAP kinase pathway, including p38 and JNK. HFD enhances JNK phosphorylation, which then phosphorylates IRS-1 at serine 307, and inhibits insulin signaling at the IRS-1 level. IKK and JNK reportedly have different roles: JNK activation is mainly related to leptin resistance to food intake, while IKK activation is more related to glucose metabolism, at least in AGRP neurons [54].

Another pathway by which SFA induces hypothalamic insulin resistance is the ceramide–PKC pathway. SFA increases the plasma membrane association of PKCθ in the hypothalamus, which inhibits insulin signaling [67]. On the other hand, inhibiting de novo ceramide synthesis, as well as PKCζ inactivation in hypothalamic neurons, normalize insulin signaling [71,72].

Reactive oxygen species (ROS) have dual roles in hypothalamic insulin signaling. Hypothalamic insulin triggers the transient production of ROS to enhance insulin signaling [73]. Activation of NADPH oxidase [73] and the mitochondrial respiratory chain [74] are reportedly mechanisms by which insulin produces ROS as the signaling molecule. In contrast, obesity and/or diabetes are related to ROS overproduction, which in turn induces inflammation, thereby blunting insulin signaling [75].

## 5. Involvement of Phosphatases and SOCS3 in Hypothalamic Insulin Resistance

Tyrosine phosphorylation of IR is the first step in insulin signaling. Protein phosphatase 1B (PTP-1B) and T-cell protein tyrosine phosphatase (TCPTP) are two major phosphatases involved in the regulation of hypothalamic insulin signaling via dephosphorylation of IR tyrosine residues [53]. PTP-1B dephosphorylates IR and IRS proteins at their tyrosine residues, and thus negatively regulates insulin signaling [76]. Hypothalamic PTP-1B expression increases in the hypothalamus of rats fed HFD for 2–4 months [77,78]. TNF-α upregulates PTP-1B expression in the hypothalamus [78], indicating chronic inflammation as the mechanism inducing PTP-1B upregulation. On the other hand, POMC-specific deletion of PTP-1B and PTP-1B knockdown in the hypothalamus by antisense oligonucleotide protects animals from HFD-induced obesity and insulin resistance [51,77]. Moreover, insulin sensitivity improved in POMC-specific PTP-1B knockout mice without changes in body weight, suggesting the impact on insulin sensitivity as the primary effect. TCPTP was also upregulated in the hypothalamus over three months of HFD feeding [52]. Neuron-specific deletion of TCPTP protects mice from developing HFD-induced obesity [52]. Its deletion in AGRP neurons enhanced the suppression of hepatic glucose production and glucose uptake in brown adipose tissue [79]. PTP-1B and TCPTP increases are initially observed after six and nine weeks of HFD feeding, respectively, suggesting that their upregulation does not trigger hypothalamic insulin resistance, which can be observed from day 1 of HFD feeding. However, these phosphatases contribute to the maintenance of hypothalamic insulin resistance.

The phosphatase and tensin homolog in chromosome 10 (PTEN) is a phosphatase that mainly dephosphorylates PIP3 and antagonizes PI 3-kinase. Constitutive activation of hypothalamic PTEN induces weight gain and insulin resistance, mimicking HFD feeding. In contrast, suppression of hypothalamic PTEN by overexpressing its dominant-negative form suppresses food intake, while this effect is blunted by HFD feeding [22]. However, even in HFD-fed animals, hypothalamic PTEN suppression reversed HFD-induced insulin resistance. During pregnancy, hypothalamic PTEN is less inactivated by low phosphorylation than during non-pregnancy periods, thereby inducing hypothalamic insulin resistance, which protects animals from insulin-induced anorexia [80]. To our knowledge, there have been no reports describing whether HFD feeding induces any changes in PTEN expression and/or its phosphorylation level.

Suppressor of cytokine signaling 3 (SOCS3) is another molecule responsible for hypothalamic insulin signaling induced by obesity. When leptin binds to its own receptor, the Janus-activated kinase-2 (JAK2)/signal transducer and activator of transcription 3 (STAT3) pathway is activated, and ultimately increases SOCS3 transcription. SOCS3 in turn suppresses insulin signaling by binding to and enhancing the degradation of IRS proteins. SOCS3 also suppresses tyrosine phosphorylation. Obesity-induced hyperleptinemia enhances SOCS3 expression, resulting in hypothalamic insulin resistance. Interestingly, the deletion of SOCS3 in leptin receptor-expressing cells protects mice from HFD-induced systemic insulin resistance, without significant weight changes [81]. This observation suggests that SOCS3 is a negative regulator of hypothalamic insulin signaling only for glucose metabolism, and not for food intake regulation.

## 6. Involvement of the mTOR-S6 Kinase Pathway in Hypothalamic Insulin Resistance

mTOR and its downstream effector, p70 S6 kinase (S6K), are activated by chronic hyperinsulinemia and excess nutrition. mTOR/S6K pathway activation leads to phosphorylation of IRS-1 at serine residues, inducing negative feedback inhibition of insulin signaling. Systemic deletion of S6K protects mice from diet-induced obesity and insulin resistance [82]. We reported that one-day HFD feeding induced S6K activation, downregulation of IRS-1 tyrosine phosphorylation, and downregulation of Akt phosphorylation in the rat hypothalamus [46]. Constitutive activation of hypothalamic S6K using viral vectors induces hypothalamic and systemic insulin resistance. Conversely, hypothalamic mTOR/S6K pathway suppression reverses HFD-induced insulin resistance. These changes in glucose metabolism are independent of body weight changes. Interestingly, the role of S6K in food intake does not parallel its effect on glucose metabolism. In contrast to its negative effect on glucose metabolism, the hypothalamic mTOR/S6K pathway suppresses food intake, which appears to be a mechanism by which the hypothalamus senses how nutrition inhibits food intake, independently of its role in the negative feedback input to insulin signaling [83].

Several reports have focused on the contradictory roles of the hypothalamic mTOR/S6K pathway. Overexpression of DEPTOR (DEP-domain containing mTOR-interacting protein), a negative regulator of the mTOR/S6K pathway, specifically using a viral vector in the mediobasal hypothalamus, prevents HFD-induced obesity and improves glucose metabolism [84]. This is consistent with our glucose metabolism observations [46], but not with reports showing that hypothalamic mTOR/S6K inhibits food intake [85,86]. Genetic deletion of S6K in POMC neurons does not affect food intake or weight, and surprisingly induces insulin resistance [87], which contradicts the results obtained in postnatal overexpression models using viral vectors [46]. Insulin resistance reported in Reference [87] was measured using a hyperinsulinemic–euglycemic clamp in anesthetized mice, where endogenous glucose production (EGP) was not fully suppressed by ~3 mU/kg/min of insulin. In clamp studies performed on non-anesthetized and non-restrained mice [88,89] or rats [46] using arterial catheter blood sampling, this level of insulin infusion was usually sufficient to completely suppress endogenous glucose production, suggesting that anesthesia-induced hepatic insulin resistance might have altered the physiological hypothalamic effect of insulin on glucose metabolism [87]. Another study showed that transgenic overexpression of DEPTOR in POMC neurons did not lead to weight changes, instead it induced slight insulin resistance [90]. These seemingly contradictory reports indicate that (1) POMC is not the main neuronal cell type mediating mTOR/S6K signals that regulate food intake or glucose metabolism, and that (2) the genetic phenotype and postnatal intervention models do not match, presumably due to the prolonged effects during the developmental period in the former. The inconsistency between a POMC-specific genetic model and postnatal intervention in the hypothalamus has also been observed for PTEN. While postnatal suppression of hypothalamic PTEN using a viral vector suppresses food intake [22]—consistent with the theory that hypothalamic insulin suppresses food intake via the PI 3-kinase pathway—the genetic deletion of PTEN in POMC neurons tends to induce weight gain instead [91]. Similarly, postnatal deletion of the leptin receptor in AGRP neurons produced very different phenotypes when compared to genetic knockout models [92,93].

ATF4 (activating transcription factor 4) is reportedly an ER stress-responsive target, which induces leanness and enhanced insulin sensitivity when deleted [94]. Hypothalamic overexpression of ATF4 induces hepatic insulin resistance, which is reversed by the inhibition of hypothalamic S6K. On the other hand, suppression of hypothalamic ATF4 reverses ER stress-induced hepatic insulin resistance. This report shows that the hypothalamic ATF4–S6K pathway is responsible for ER stress-induced hypothalamic insulin resistance, which results in hepatic insulin resistance. A chemokine, CCL5/RANTES, activates CCR5 and reportedly decreases serine phosphorylation of IRS-1 in the hypothalamus by suppressing S6K [95]. Blocking CCL5/RANTES–CCR5 by genetic deletion or ICV injection of an antagonist inhibited hypothalamic insulin signaling and insulin resistance, indicating that this chemokine has a role in suppressing S6K-mediated negative feedback input to insulin signaling.

## 7. Cell Populations Involved in Hypothalamic Insulin Functions and Insulin Resistance

Insulin has been detected in the CSF at concentrations 10–25% of those in the bloodstream. When plasma insulin increases, CSF insulin levels also rise [96]. While some brain insulin may be synthesized in the CNS [97], most is thought to come from the bloodstream [98]. Obesity increases CSF insulin levels in rodents [47], sheep [48], and humans [49]. However, the transport ratio of insulin from the periphery to the brain is blunted by HFD feeding [99,100]. Insulin-resistant individuals have a lower CSF/blood insulin concentration ratio [101]. Therefore, hypothalamic insulin resistance is partly explained by disrupted insulin transport into the brain. A portion of insulin in the brain is transported from the bloodstream into the brain by IR-expressing brain endothelial cells [14,38,96,98]. Insulin transport by brain endothelial cells reportedly decreases by HFD feeding [12], while insulin transport via receptor-mediated endothelial transcytosis remains controversial [96]. IR deletion in astrocytes blunted insulin transport into the brain, demonstrating that astrocytes are also involved in this transport [10,11].

In addition to neurons, glial and brain endothelial cells are also present in the hypothalamus, where research has been focused on determining which cell types are involved in insulin functions and hypothalamic insulin resistance [102,103]. IR expression levels are higher in neurons compared to glial cells. Among neuronal cells, AGRP-expressing neurons are the primary site in which insulin acts to suppress hepatic glucose production [33,79,104]. POMC-expressing cells are responsible for the central insulin functions of suppressing lipolysis and promoting lipogenesis in adipose tissue [33]. However, the role of POMC neurons in suppressing glucose production is controversial. POMC-specific genetic deletion of IR does not significantly affect the ability of insulin to suppress glucose production [33]. It was recently found that TCPTP expression is upregulated by fasting and downregulated by feeding, and this upregulation of TCPTP in POMC neurons during fasting masks the suppressive effects of insulin on glucose production [105]. When TCPTP expression in POMC neurons is suppressed by genetic deletion or feeding, the ability of insulin to suppress glucose production becomes apparent. Non-AGRP-expressing, neuropeptide Y (NPY)-expressing neurons have been recently shown to be responsible for the suppressive action of insulin on food intake [106].

Cultured hypothalamic neuronal cells are resistant to SFA-induced inflammation and insulin resistance [107], suggesting that SFA mainly affects non-neuronal cells, leading to neuronal insulin resistance. HFD feeding for one day is enough to induce hypothalamic gliosis, including both microgliosis and astrogliosis [108]. Microgliosis is induced by HFD feeding, but not obesity [63], and contributes to hypothalamic inflammation [62,109]. In contrast, astrogliosis is recognized as a protective reaction of the brain responding to acute excess nutrition [110]. The roles of inflammation, including that of the IKKβ/NFκB pathway in astrocytes, are controversial, because while one report has shown that the inhibition of NFκB in astrocytes enhanced food intake [111], another demonstrated astrocytic-mediated inhibition of NFκB to protect animals from HFD-induced obesity [112,113]. Astrocyte-specific IR deletion was shown to disturb glucose sensing, in addition to insulin and glucose transport from the bloodstream into the brain. The animal model used also showed impaired glucose tolerance and insulin resistance [11]. Brain endothelial cells are another cell type responsible for the transport of circulating insulin into the brain, where the uptake of insulin was downregulated after weeks of HFD feeding, showing an increase in NFκB binding activity [12]. Insulin transport by brain endothelial cells is not dependent of PI3k signaling, and the mechanism of the insulin resistance in brain endothelial cells induced by HFD feeding merits further study.

Long-term hypothalamic inflammation results in hypothalamic angiogenesis [114] and loss of POMC neurons. Moreover, long-term HFD induces expansion of the macrophage pool, which normally resides in the median eminence, to the arcuate nucleus of the hypothalamus. Inhibition of inducible nitric oxide synthase in these hypothalamic macrophages not only abrogates macrophage activation, but also improves glucose metabolism [115].

## 8. Concluding Remarks

Given the current global pandemic of obesity and related diseases such as type 2 diabetes, and the lack of effective treatments, understanding the molecular mechanisms of hypothalamic insulin resistance is necessary for the development of safe and efficacious medications for the treatment of these metabolic disorders. Insulin nasal sprays effectively stimulate hypothalamic insulin signaling to suppress food intake as well as glucose production in lean but not obese men. Thus, the same approach might be useful for administering novel drugs that reverse or bypass the blockage point of insulin signaling as an obesity treatment, once the blockage mechanism and the responsible molecules have been fully elucidated. We now know that numerous heterogeneous cell types in the hypothalamus (POMC or AGRP neurons, other neurons, astrocytes, microglia, endothelial cells, macrophages in median eminence, etc.) are related to hypothalamic insulin resistance. Since commonly used drugs are not effective exclusively in one cell type, even one specific to the hypothalamus, when administered nasally they might not exert the desired “total effect” on heterogeneous hypothalamic cells. Thus, each candidate medication should be carefully studied and considered before its application to patients.

## Abbreviations

AchR	Acetylcholine receptor
AGRP	Agouti-related protein
ATF4	Activating transcription factor 4
BAT	Brown adipose tissue
BBB	Blood–brain barrier
CCL5	C C motif chemokine 5
CCR5	C–C chemokine receptor type 5
CpE	Carboxypeptidase E
CSF	Cerebrospinal fluid
DEPTOR	DEP domain-containing mTOR-interacting protein
DREADD	Designer receptors exclusively activated by designer drugs
EGP	Endogenous glucose production
ER stress	Endoplasmic reticulum stress
FoxO1	Forkhead box protein O1
G6Pase	Glucose 6-phosphatase
GPR	G protein-coupled receptor
HFD	High-fat diet
IκB	Nuclear factor of kappa-light-chain-enhancer in B-cells inhibitor
IKKβ	IκB kinase β
IL-6	Interleukin 6
IR	Insulin receptor
IRS	Insulin receptor substrate
JNK	c-Jun-NH2-terminal kinase
mTORC1	Mechanistic target of rapamycin complex 1
Myd88	Myeloid differentiation primary response 88
NFκB	Nuclear factor of kappa-light-chain-enhancer in B-cells
NPY	Neuropeptide Y
PDE3B	Phosphodiesterase 3B
PEPCK	Phosphoenolpyruvate carboxykinase
PI3K	Phosphoinositide 3-kinase
PIP3	Phosphatidylinositol 3,4,5-triphosphate
PKC	Protein kinase C
POMC	Proopiomelanocortin
PTEN	Phosphatase and tensin homolog on chromosome 10
PTP-1B	Protein tyrosine phosphatase 1B
RANTES	Regulated on activation, normal T cell expressed and secreted
Rictor	Rapamycin-insensitive companion of mammalian target of rapamycin
ROS	Reactive oxygen species
S6K	P70 S6-kinase
SFA	Saturated fatty acids
STAT3	Signal transducer and activator of transcription 3
TCPTP	T-cell protein tyrosine phosphatase
WAT	White adipose tissue
